# The Influence of *Drosophila* Spire and Myosin V During Mid‐Oogenesis Is Independent of Their Direct Interaction

**DOI:** 10.1002/cm.70080

**Published:** 2025-12-08

**Authors:** Joseph Y. Ong, Hannah M. Bailey, Emma Carley, Michelle Panzica, Merin Rixen, Neil Billington, Rong Liu, Margot E. Quinlan

**Affiliations:** ^1^ Department of Chemistry and Biochemistry University of California Los Angeles California USA; ^2^ Department of Biochemistry and Molecular Medicine West Virginia University Morgantown West Virginia USA; ^3^ Molecular Biology Institute University of California Los Angeles California USA

**Keywords:** actin, *Drosophila*, myosin V, oogenesis, spire

## Abstract

Cooperativity between cytoskeletal proteins is crucial for spatiotemporal coordination in biological processes, like oogenesis. In mammalian and *Drosophila* oogenesis, proper assembly and function of actin networks require coordination between actin assembly factors Spire and formins, as well as actin‐associated proteins like myosins and Rab GTPases. Here, we investigate the interaction between Spire and Myosin V (MyoV) in *Drosophila* oogenesis. We combine in vitro biochemical assays with immunofluorescence and genetics to probe the interaction and its impact on polarity establishment and the actin mesh that fills the oocyte during mid‐oogenesis. Expressed Spire and MyoV constructs colocalize in punctae during mid oogenesis, with considerable enrichment near the oocyte cortex. Purified constructs interact directly in vitro, and we find that Spire can weakly activate MyoV ATPase activity. Cytoplasmic flows, critical for polarity establishment, are faster and more coordinated in the absence of MyoV, although not to the extent of fast streaming. This intermediate streaming has not been observed before. Interestingly, this MyoV‐dependent change in ooplasm dynamics is sensitive to Spire levels. Despite this interplay, the actin mesh and polarity establishment appear normal when binding mutants of Spire and MyoV are expressed in the *Drosophila* germline. These findings suggest that direct interaction between Spire and MyoV is not essential for their primary roles at this stage of development.

## 1 Introduction

The cytoskeleton plays critical roles at essentially every step of oogenesis. A great amount of our knowledge comes from work in the classical model organism, *Drosophila melanogaster* (Hudson and Cooley 2002; Robinson and Cooley 1997; Rodal et al. 2015). In more recent years, advances in our understanding of the cytoskeleton in both *Caenorhabditis elegans* and mammalian (mouse, pig, and human) oogenesis demonstrate that many structures and processes are conserved across species (Cheng et al. 2022; Harasimov et al. 2023). For example, a dynamic actin mesh that fills the developing oocyte, in all of these species, is required for proper development (Azoury et al. 2008; Dahlgaard et al. 2007; Panzica et al. 2017; Quinlan 2013; Schuh and Ellenberg 2008). In the mouse and the fly, this mesh is built by a pair of actin nucleators, Spire (Spire‐1 and Spire‐2 in mammals; Spir in the fly) and Fmn‐family formins (Fmn‐2 but not Fmn‐1 in mammals; Capu in the fly) (Azoury et al. 2008; Dahlgaard et al. 2007; Pfender et al. 2011; Schuh and Ellenberg 2008). The *spire* and *fmn* genes were lost from the *C. elegans* genome. However, treatment with the pan‐formin inhibitor, SMIFH2, and RNAi of profilin leads to loss of mesh and sperm DNA mislocalization, suggesting that a different formin builds the mesh in this species (Panzica et al. 2017). In the mouse oocyte, Spire‐1/2 and Fmn‐2 colocalize with Myosin V (MyoV) on punctae throughout the mesh (Holubcová et al. 2013; Pfender et al. 2011). Interplay between actin turnover and MyoV motor activity creates symmetric active diffusion which maintains the nucleus in the center of the spherical oocyte during metaphase (Almonacid et al. 2015; Azoury et al. 2011; Azoury et al. 2008). In fact, the mesh density changes throughout development suggesting multiple, stage‐dependent roles for the mesh, including roles in nuclear positioning and long‐distance movement of Rab‐11 positive vesicles (Azoury et al. 2011; Holubcová et al. 2013; Schuh 2011). The *Drosophila* actin mesh also has multiple roles in oogenesis. It helps maintain microtubule organization, which provides tracks for polarity determinant transport (Dahlgaard et al. 2007). In addition, the dynamics of the mesh contribute directly to this transport process (Drechsler et al. 2017). The dynamics of the ooplasm are described by the terms slow streaming or seething when the mesh is present; in the absence of the mesh, kinesin‐driven motion leads to microtubule rearrangement and fast streaming throughout the oocyte (Dahlgaard et al. 2007; Palacios and Johnston 2002; Serbus et al. 2005; Theurkauf 1994). Both the presence of the mesh and its timely removal are important for *Drosophila* oogenesis (Bor et al. 2015; Dahlgaard et al. 2007; Quinlan 2013).

MyoV was the first processive actin‐based motor discovered (Mehta 2001; Mehta et al. 1999; Sakamoto et al. 2000). It can take many steps along actin filaments while transporting cargo. The cargo varies; they include, for example, melanosomes in dendritic cells and melanocytes, pigment granules in photoreceptors, secretory granules in neurons, and more (Hammer and Sellers 2012; Wong and Weisman 2021). Vertebrates have three classes of MyoV (MyoVa, Vb, and Vc); specificity of function is determined, in part, by cargo‐binding adapters (Pylypenko et al. 2013; Wei et al. 2013). The first adapter identified was melanophilin (Fukuda and Kuroda 2002). While melanophilin is sufficient to activate MyoVa's actin‐activated ATPase activity, to reconstitute active movement of cargo, a tripartite complex between MyoVa, melanophilin, and Rab27 is required (Li et al. 2005; Wu et al. 2006). In the *Drosophila* photosensor, an analogous complex is formed between MyoV, Rab11, and dRip11 (Li et al. 2007), suggesting that other proteins function in roles similar to melanophilin.

Spir and MyoV colocalize on punctae that are part of the actin mesh in mouse oocytes (Holubcová et al. 2013). In addition, a direct interaction between mammalian Spir (the so‐called MyoV Globular Tail Binding Motif, GTBM) and MyoV (Globular Tail Domain, GTD) was described (Pylypenko et al. 2016). Notably, the Spir‐binding site on the MyoV‐GTD is very similar to the melanophilin binding site (Pylypenko et al. 2016, 2013; Wei et al. 2013). In addition, the data suggest that Spir and Rab11 can simultaneously bind MyoV (Pylypenko et al. 2016). In melanocytes, data indicate that Spir, Rab27, and MyoV collaborate to move melanosomes (Alzahofi et al. 2020). Finally, recent in vitro studies demonstrate that Spir2 and Rab11a work together to activate MyoVb (Yao et al. 2024). Together these data support a model in which Spir is a MyoV‐adaptor protein, similar to melanophilin.

*Drosophila* have only one MyoV gene (*didum*) to play these disparate roles. Perdurance of MyoV in the germline originally muddied interpretations of phenotypes; a role for MyoV in *Drosophila* spermatid individuation was observed but its role in oogenesis was obscured (Mermall et al. 2005). A role for MyoV in the *Drosophila* oocyte was first established in a screen for maternal‐effect genes involved in patterning (Krauss et al. 2009; Luschnig et al. 2004). This group found that while progeny derived from *didum* germline clones (GLCs; Figure S1) can be used to study MyoV, the phenotype was strongest in GLCs when induction occurred during early larval development, consistent with residual MyoV activity in the oocytes masking phenotypes and more so in mature oocytes (Krauss et al. 2009). MyoV is important for posterior enrichment of Staufen/oskar (Krauss et al. 2009). The motor is not a part of oskar RNPs, ruling out a direct role in transport. Instead, Lu et al. (2018) observed that MyoV is necessary to slow particles at the posterior, presumably facilitating capture and/or anchoring of oskar RNPs. MyoV is found throughout the oocyte cortex, leading one to question how it works specifically at the posterior end of the cell (Krauss et al. 2009; Lu et al. 2018). One major difference at this end of the oocyte is the absence of microtubules growing from the cortex (Nashchekin et al. 2016; Parton et al. 2011; Shulman et al. 2000; Zimyanin et al. 2008). Thus a model was proposed in which a tug‐of‐war between MyoV/actin motility and kinesin/microtubule motility exists everywhere along the cortex except at the posterior (Lu et al. 2020). Analogous competition between kinesin and MyoV is observed in multiple cell types (Pathak et al. 2010; Rogers and Gelfand 1998; van Bergeijk et al. 2015; Wu et al. 1998).

Premature fast streaming, loss of the actin mesh, loss of polarity, and infertility are well‐described phenotypes of *spir* mutants (Dahlgaard et al. 2007; Manseau and Schupbach 1989; Theurkauf 1994; Wellington et al. 1999). Loss of MyoV function (*didum*‐GLCs and dominant negative overexpression of MyoV fragments) also results in polarity defects and decreased fertility, though not as severe (Krauss et al. 2009; Luschnig et al. 2004). Therefore, we asked whether the Spir‐MyoV interaction is important for *Drosophila* oogenesis. We found that the two proteins do interact but with relatively low affinity. We report direct binding in vitro and colocalization in vivo. However, our data suggest that the direct link is not necessary for oogenesis. Instead, the experiments led to the discovery of an intermediate state between slow and fast streaming, suggesting a role for MyoV as a crosslinker in the actin mesh. Of note, *Drosophila* MyoV is not a processive motor, at least in vitro (Bonafe and Sellers 1998). Perhaps adapter proteins alter the biochemical cycle or hold MyoV close enough to actin to make it more processive. Alternatively, MyoV may be specialized to anchor, as opposed to transport, in the fruit fly.

## 2 Results

### 2.1 Spir‐MyoV Interaction Is Conserved in *Drosophila*

While the MyoV‐binding sequence in Spir is well‐conserved in vertebrates, the same region of the *Drosophila* Spir sequence does not align well (Pylypenko et al. 2016). However, a region in Spir with weak similarity to the vertebrate MyoV binding site (GTBM) aligns well with melanophilin, which binds MyoV in a closely overlapping site (Figure 1A,B) (Pylypenko et al. 2016; Wei et al. 2013). This sequence as well as the target MyoV‐GTD is highly conserved among insect isoforms of Spir, suggesting that the Spir/MyoV interaction could be preserved in insects (Figure 1C,D). Given that Spir and MyoV play important roles in mammalian and *Drosophila* oogenesis, we asked if the proteins interact in *Drosophila* oocytes.

**FIGURE 1:**
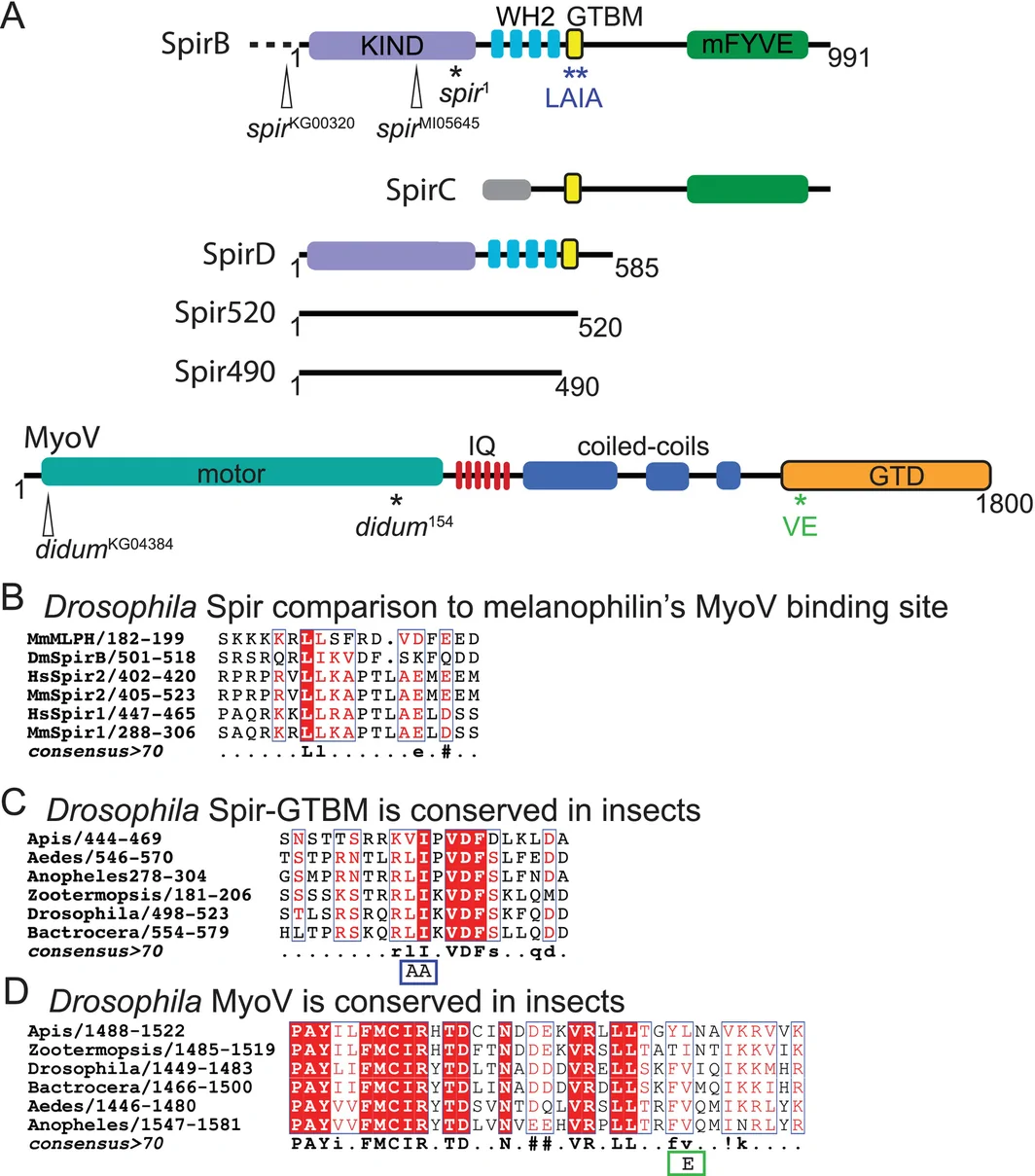
Spir‐MyoV binding sites are conserved in *Drosophila*. (A) Cartoons depicting the major known domains within Spir and MyoV are shown. Indicated domains are: GTBM, (MyoV‐) globular tail binding motif; GTD, globular tail domain (which includes the Spir binding site; IQ, isoleucine (I) glutamine (Q) motifs known to bind calmodulin‐like light chains; KIND, Kinase INactive Domain; mFYVE, modified FYVE domain; WH2, Wasp homology 2 domain). Transposon insertion sites are indicated by triangles. Mutations made in this paper have color‐coded asterisks and their nomenclature below (blue, LAIA: L507A, I508A; green, VE: V1477E). Additional alleles used are labeled with black asterisks and their common names. Spir constructs used are indicated below the full length protein. (B) *Drosophila* Spir's putative GTBM aligns well with the MyoV binding site in melanophilin. (C) The GTBM is highly conserved in insects (D) as is the Spir binding region within the MyoV‐GTD. The residues boxed below the alignments in (C) and (D) indicate the mutations made (Spir‐LAIA and MyoV‐VE). Alignments created with Clustal Omega and ESPript 3 (Gouet et al. 1999; Sievers et al. 2011).

First, we re‐examined the localizations of Spir and MyoV in ovaries. To do so, we expressed MyoV‐GFP and SpirC‐3xHA in ovaries (Bischof et al. 2013; Krauss et al. 2009). SpirC is a splice variant of Spire that contains 93 unique N‐terminal amino acids followed by the SpirB sequence from amino acid 491 to the C‐terminus (Figure 1A). We used it as a proxy for SpirB because it can be expressed at higher levels than full‐length Spir and is less deleterious to egg chambers when overexpressed. As expected, SpirC‐3xHA (Figure 2A) and MyoV‐GFP (Figure 2B) are enriched at the oocyte cortex (see also Figure S2A–C) (Krauss et al. 2009; Lu et al. 2020; Quinlan 2013). When both MyoV‐GFP and SpirC‐3xHA are expressed in the same cell, they colocalize, similar to what is seen in the mouse oocyte (Figure 2D) (Holubcová et al. 2013). When examined at higher spatial resolution, we observed punctae positive for both SpirC‐3xHA and MyoV‐GFP at the cortex and in the cytoplasm (Figure 2E). Punctae with only one protein were also observed, which could reflect mechanism and/or expression levels. In general, MyoV‐GFP appeared more continuous than SpirC‐3xHA along the cortex (Figure 2E′,E″). We also measured higher levels of MyoV‐GFP at the posterior of the oocyte relative to the lateral cortex (Figure 2B,C). While posterior enrichment was not reported previously, it is robust in our hands and the enrichment is consistent with its role in posterior anchoring (Krauss et al. 2009; Lu et al. 2020, 2018).

**FIGURE 2:**
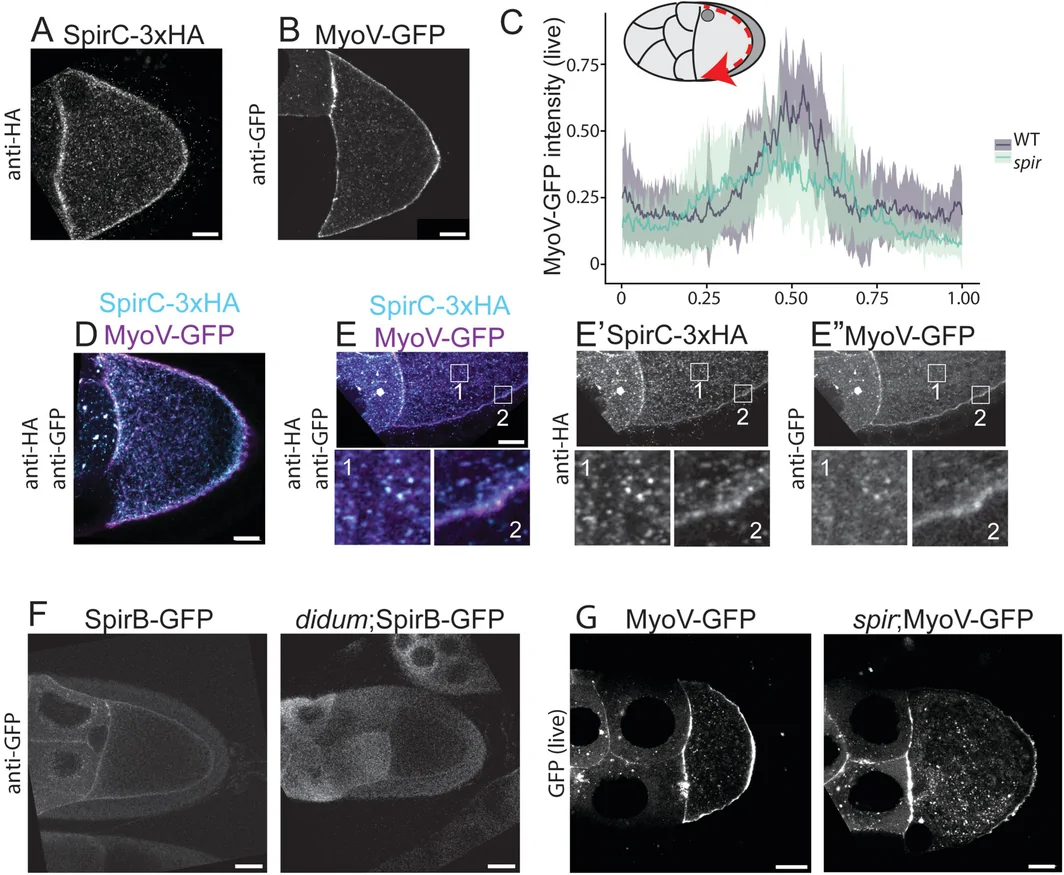
Localization of Spir and MyoV in *Drosophila* oocytes. (A) SpirC‐3xHA is enriched in the cortex of a stage 9 oocyte. nos::GAL4 > UASg::SpirC‐3xHA is expressed and the HA tag is stained. (B) MyoV‐GFP is enriched in the cortex of a stage 9 oocyte. nos::GAL4 > UASp::MyoV‐GFP is expressed and GFP is stained. (C) In live flies, GFP intensity along the cortex reveals enrichment of MyoV‐GFP at the posterior of the oocyte in a wild‐type (WT) background. The bias is decreased in a *spir* background (for WT, *n* = 10 oocytes from four flies; for *spir*, *n* = 10 oocytes from three flies). (D) SpirC‐3xHA and MyoV‐GFP colocalize in a stage 9 oocyte. See Figure S2A–C for negative controls. (E) Higher magnification of the side of an oocyte expressing SpirC‐3xHA and MyoV‐GFP shows punctae, positive for both proteins, in the cytoplasm (box 1) and along the cortex (box 2). (E′ and E″) Separate channels for SpirC‐3xHA and MyoV, respectively. (F) Comparison of SpirB localization in wild type versus *didum* backgrounds. Localization of SpirB‐GFP matches that of SpirC‐3xHA in a wild type background. GFP staining shows protein enriched at the cortex and punctae throughout the cytoplasm. Enrichment of SpirB‐GFP at the cortex is lost in a *didum* background. nos::GAL4 > UASp::SpirB‐GFP is expressed and GFP is stained. See Figure S2D,E for further analysis. (G) Direct observation of GFP shows that MyoV‐GFP in live egg chambers localizes as seen in fixed samples (B). In a *spir* background, MyoV‐GFP localization is only mildly altered. The posterior enrichment is decreased (see C). Scale bars = 20 μm.

Based on their colocalization, we next asked if Spir and MyoV localizations were interdependent. In order to remove MyoV from the germline, we built *didum*‐GLCs using *didum* ^*154*^ (see Figure S1 for more detail). These *didum* ^154^‐GLCs are not nulls. Krauss et al. (2009) demonstrated that *didum* ^154^‐GLC egg chambers have a small amount of residual full‐length MyoV (see Krauss et al. 2009; Figure S1E). In addition, phenotypic differences observed in the original report and here are consistent with the expression of a stable product from *didum* ^*154*^ in GLCs, which would include most of the MyoV motor domain. Thus *didum* ^154^‐GLCs are loss‐of‐function egg chambers with a dominant negative protein fragment present. Importantly, the fragment does not include the Spir binding site. In order to perform functional assays, in addition to examining localization, we expressed SpirB‐GFP in the following experiments. Like SpirC‐3xHA, SpirB‐GFP punctae were enriched at the cortex and visible throughout wild‐type oocytes (Figure 2F). However, in *didum* ^154^‐GLCs the staining of SpirB‐GFP along the cortex was only occasionally detected, and significantly weaker when quantified as a function relative to oocyte intensity (1.8 ± 0.3 vs. 1.3 ± 0.3, *p* = 0.008 Student's two‐tailed *t*‐test) (Figure 2F, Figure S2D,E). Furthermore, we could not detect punctae at the cortex or in the cytoplasm in these ovaries. The variability may be due to some MyoV perdurance in GLCs (Krauss et al. 2009). Given that SpirB‐GFP expression is readily detected in the nurse cells in both backgrounds, the results suggest that MyoV drives and/or stabilizes Spir localization in the oocyte punctae.

The distribution of exogenously expressed MyoV‐GFP was essentially indistinguishable in wild‐type and *spir*/+ oocytes. In *spir*‐null oocytes, however, we observed a decrease in posterior enrichment of MyoV‐GFP (Figure 2C,G). A well‐described phenotype of *spir*‐null oocytes is the premature onset of rapid cytoplasmic flows, which could be responsible for the difference (Dahlgaard et al. 2007; Theurkauf 1994). We, therefore, examined MyoV‐GFP in *capu*‐null oocytes, which also exhibit premature cytoplasmic flows (Dahlgaard et al. 2007; Theurkauf 1994). Loss of posterior enrichment of MyoV‐GFP was similar in most *capu*‐null oocytes (11 eggs from six flies = set 1) but all of the eggs examined from a single fly looked wild type (set 2) (Figure S2F,G). Thus, at this time, we cannot determine whether decreased posterior enrichment of MyoV is an indirect effect, due to mechanical disruption caused by premature streaming, or a more direct effect, due to loss of Spir and/or the actin mesh it builds with Capu. If the MyoV loss from the posterior is due to streaming, as opposed to the absence of Spir, it suggests that there are two populations of MyoV at the posterior: one stably associated with the cortex and another that is not, which may explain why MyoV enrichment was not previously observed.

### 2.2 *Drosophila* Spir Directly Binds MyoV

To test whether colocalization reflects interaction, we asked whether Spir and MyoV co‐immunoprecipitate. We combined ovary lysates from flies expressing SpirC‐3xHA or Rab11‐3xHA (as a positive control) with lysates from flies expressing MyoV‐GFP or GFP. MyoV‐GFP but not GFP immunoprecipitated with both Rab11‐HA and 3xHA‐SpirC (Figure 3A and Figure S3C). These data suggest that MyoV and Spir bind to each other in the *Drosophila* oocyte.

**FIGURE 3:**
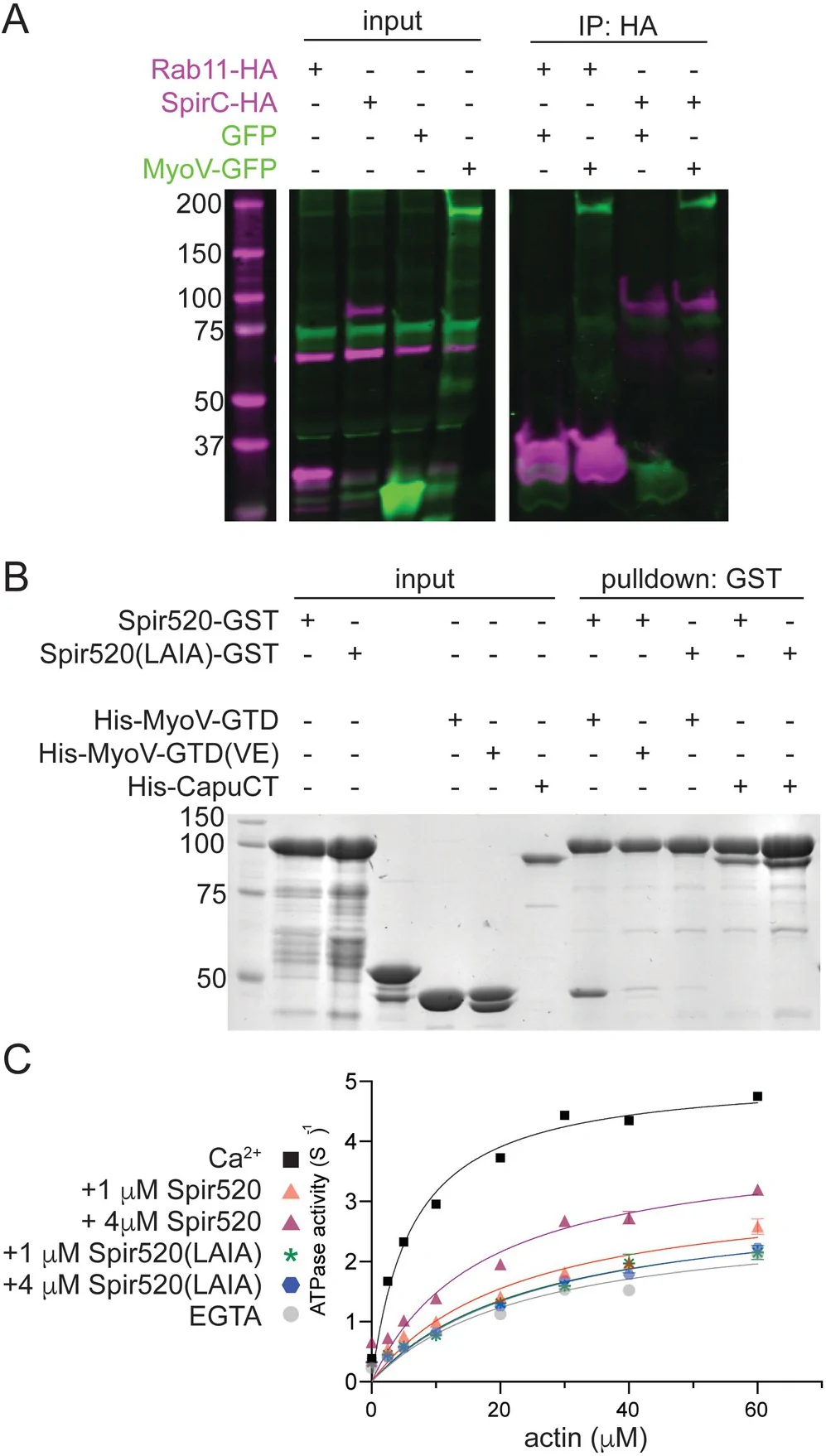
*Drosophila* Spir directly binds MyoV. (A) Anti‐HA immunoprecipitation from ovary lysates. Lysates of ovaries expressing SpirC‐3xHA or Rab11‐3xHA were mixed with MyoV‐GFP or GFP containing lysates. MyoV‐GFP co‐IPs with both SpirC‐3xHA and Rab11‐3xHA in the complex lysate. GFP alone is not immunoprecipitated (see Figure S3C for anti‐HA control). (B) GST‐pull downs of purified proteins. Spir520‐GST pulls His‐MyoV‐GTD down but point mutations in either Spir or MyoV diminishes the interaction. Pull down of His‐CapuCT is shown as a positive control. (C) Steady‐state ATPases assays with indicated concentrations of actin. Spir520 partially activates MyoV ATPase activity but Spir520(LAIA) does not.

Using a yeast two‐hybrid assay, we determined that Spir490‐520 (Figure S3A,B), which contains the conserved motif shown in Figure 1B, is necessary for interaction with the MyoV‐Globular Tail Domain (GTD, aa 1414–1800). We then expressed and purified Spir520‐GST and MyoV‐GTD constructs to probe for a direct interaction. Indeed, purified Spir520‐GST pulls down MyoV‐GTD (Figure 3B). We also generated and purified constructs with point mutations: Spir520(LAIA)‐GST (LAIA: L507A, I508A) and MyoV‐GTD(VE) (VE: V1477E). Consistent with structural and biochemical studies (Pylypenko et al. 2016, 2013; Wei et al. 2013), Spir520(LAIA)‐GST and MyoV‐GTD(VE) binding to their respective wildtype counterparts is greatly diminished in pull‐down assays (Figure 3B). Thus, we have confirmed that the Spir/MyoV interaction described in mammals is conserved in *Drosophila*.

Next, we asked if the interaction alters the biochemical activities of either protein. The MyoV binding partner, melanophilin, is sufficient to activate MyoV (Li et al. 2005). Because Spir binds a closely overlapping site, we measured its ability to activate the steady‐state actin‐activated ATPase activity of full‐length *Drosophila* MyoV. Spir520 activated MyoV's enzyme activity in a dose‐dependent manner (1 and 4 μM tested vs. 20 nM MyoV), whereas Spir520(LAIA) did not, under identical conditions (Figure 3C). However, even in the presence of 4 μM wild‐type Spir520, the activation remains partial, in comparison to calcium‐induced activity, consistent with a low binding affinity (see Figure S4A–C).

Spir's MyoV‐GTD binding site is adjacent to the tandem actin‐monomer‐binding domains or Wasp Homology 2 domains (WH2), which are responsible for nucleation. We, therefore, performed pyrene‐actin assembly assays with Spir520 in the presence or absence of Myo‐GTD. We observed no difference in Spir‐mediated actin assembly kinetics due to the addition of Myo‐GTD (Figure S4D).

These data demonstrate that, in the absence of other factors or protein binding partners, Spir can weakly activate MyoV, as predicted based on its binding site, but the converse effect on Spir's activity is not likely.

### 2.3 Opposing Mesh Impacts due to Decreased Spir Versus MyoV

In order to tease apart their roles, we examined the consequences of decreasing levels of Spir and/or MyoV on cytoplasmic flows and polarity establishment. Cytoplasmic streaming is described as slow (~20 nm/s) and uncoordinated motion of the ooplasm during mid‐oogenesis (before stage 10B) or fast (~300 nm/s) and highly coordinated motion beginning at stage 10B. Here, we report coordination as the maximal radius at which the motion vectors are half correlated, *C*(*r*)_0.5_. During mid‐oogenesis *C*(*r*)_0.5_ = 2.7 ± 1.8 μm and increases to 23.4 ± 10.3 after stage 10B (Figure 4A,B). Mutations in a small set of genes, including *spir* and *capu*, cause premature (i.e., before stage 10B) fast streaming with an average velocity of ~100 nm/s (here 100 ± 24 nm/s) and increased coordination (*C*(*r*)_0.5_ = 15 ± 5 μm; Figure 4B,C) (Ganguly et al. 2012; Quinlan 2013; Theurkauf 1994). We examined two alleles of *didum*: *didum* ^KG04384^, a P‐element insertion near the beginning of the coding region, resulting in a presumptive null, and *didum* ^154^, a premature stop at residue 681 which results in expression of most of the motor domain (Figure 1A) (Krauss et al. 2009). In some cases, phenotypes were more penetrant in *didum* ^154^ heterozygotes than *didum* ^KG04384^, presumably because the expressed motor domain acts as a dominant negative, exacerbating the absence of full‐length MyoV (Eppinga et al. 2008; Holubcová et al. 2013; Krauss et al. 2009). Thus we kept these data sets separate. We also examined two alleles of *spir: spir* ^MI05646^, a Mi{MIC} insertion between the second and third coding exon (after K222), and *spir* ^1^, a premature stop, E292@ (Manseau and Schupbach 1989; Venken et al. 2011). Both alleles are within the first folded domain, the Kinase INactive Domain (KIND), and result in presumptive nulls (Figure 1A) (Ciccarelli et al. 2003; Quinlan et al. 2007). We detected no differences between the two alleles and, therefore, pooled the data (labeled *spir*). No significant differences were detected in the stage 9 streaming velocities of *spir*/+, *didum* ^KG04384^/+, *didum* ^154^/+, or the two *spir* ^*1*^ /didum* transheterozygous pairs relative to wild type or each other (Figure 4B). However, the coordination of movement increased in the *didum* ^*154*^/+ background and for both *spir* ^*1*^ /didum* transheterozygotes, when compared to wild type (Figure 4C), demonstrating a subtle impact of decreasing the amount of MyoV in the oocyte, which is more apparent when the isolated motor domain is present (*didum* ^154^/+).

**FIGURE 4:**
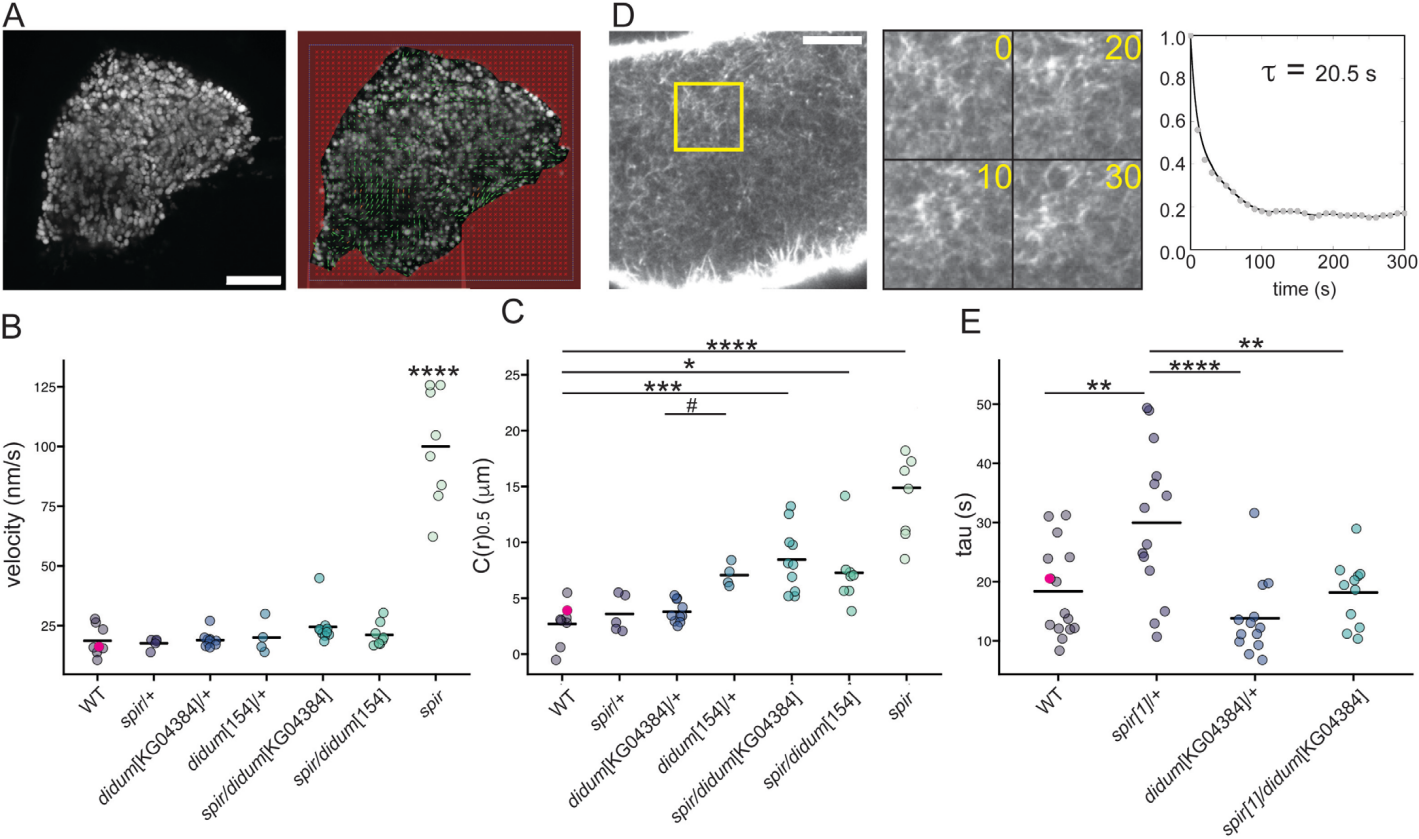
Analysis of streaming and mesh in heterozygous backgrounds. (A) Time lapse projection of yolk granule autofluorescence in a stage 9 oocyte (1 image/5 s for 5 min). (A′) Overlay of PIV analysis shows vectors that reflect velocity and are further analyzed to measure correlation of motion. Analyses of this oocyte are indicated by magenta data points in (B) and (C). (B) Streaming velocity is unchanged in heterozygotes and transheterozygotes. (C) Correlation is increased in *didum* ^154^/+, and both spir*/didum backgrounds, relative to wild type. (D) Left, Image of the actin mesh labeled with UtrnCH‐GFP in a stage 9 oocyte. Center, Zoom in on mesh (indicated with yellow box) at four timepoints (numbers are seconds). Right, Autocorrelation analysis reflects the dynamic rearrangement of the mesh. Analysis of this oocyte is indicated by a magenta data point in (E). (E) Comparison of time constants reveal that mesh dynamics are decreased in a *spir* ^1^/+ background relative to wild type. Time constants in transheterozygous oocytes, *spir* ^*1*^ */didum* ^KG04384^, are faster than *spir* ^1^/+, matching dynamics of wild type mesh (spir* indicates *spir* ^*1*^ and *spir* ^MI05646^ data combined; only *spir* ^*1*^ was tested in E). Statistical analyses are one‐way ANOVAs followed by Tukey Post Hoc tests. Significance codes: ****p < 0.0001, ***p < 0.001, **p < 0.01, *p < 0.05. ^#^Correlations of *didum* ^KG04384^/+ and *didum* ^154^/+ differ significantly, when *spir* data are excluded, *p* = 0.00013. Scale bars = 20 μm.

Cytoplasmic flows are limited by the actin mesh. We, therefore, examined the mesh in these genetic backgrounds as well. We did not detect differences in mesh density of fixed samples. However, by expressing UtrnCH‐GFP to label filamentous actin, we measured a change in mesh dynamics in live samples (Figure 4D,E). Using autocorrelation analysis, we detected a significant increase in the time constant (i.e., decrease in dynamics) of *spir* ^1^/+ (Figure 4E). Interestingly, the altered mesh dynamics do not correlate with the coordination of motion in the oocyte (*C*(*r*)_0.5_). Instead, the mesh dynamics in the *spir* ^1^/*didum* ^KG04384^ background were significantly different from those of *spir* ^1^/+, and indistinguishable from wild type. The dynamics data suggest that Spir and MyoV influence the mesh in opposing ways (while the difference is not statistically significant, there is a small increase in mesh dynamics in the *didum* ^KG04384^/+ oocytes, consistent with an apparent rescue observed in the transheterozygous oocytes).

Posterior localization of *oskar* mRNA and Staufen is disrupted in strong *spir* and *didum* mutant backgrounds (Krauss et al. 2009; Wellington et al. 1999). While we observed abnormal or missing Stau localization in *didum* ^*154*^‐GLCs and *spir*‐nulls, as previously reported, we did not observe a change in Stau in fixed stage 9 oocytes of heterozygotes (*spir*/+ or didum*/+) or *spir/didum* transheterozygotic stage 9 oocytes (Figure S5A). After observing the increase in coordination described above, we examined GFP‐Stau localization in live oocytes. Even in oocytes that had a visibly increased degree of coordination, GFP‐Stau localization at the posterior was indistinguishable from wild type (Figure S5B). Thus, the altered oocyte dynamics are not sufficient to disrupt posterior transport or anchoring. Combined with wild‐type levels of fertility in *spir/didum* transheterozygotes (Table S1), these results indicate that both Spir and MyoV are present at levels in excess of what is required for polarity establishment.

### 2.4 A Streaming Intermediate

Based on the differences in mesh dynamics and fluid flow coordination we observed in *didum* heterozygotic oocytes, we examined cytoplasmic streaming in *didum* ^154^‐GLCs. The mesh was denser than that in wild type, which is consistent with reports that *didum* defects do not cause premature streaming (Figure 5A,B) (Krauss et al. 2009). Likewise, overexpression of MyoVa tail in mouse oocytes does not lead to loss of mesh (Holubcová et al. 2013). However, the apparent velocity of yolk granules was increased (Figure 5C). Analysis revealed that velocity and *C*(*r*)_0.5_ in *didum* ^154^‐GLCs were significantly increased relative to wildtype (Figure 5D,E). Upon closer examination, we found that the streaming behavior of *didum* ^154^‐GLCs fell into two classes: about half were indistinguishable from wild type (*C*(*r*)_0.5_ = 2.6 ± 0.7 μm, class I) while the other half had a significantly higher *C*(*r*)_0.5_ (9.8 ± 3.5 μm, class II) (Figure 5E). When split according to correlation class, the streaming velocity in class II oocytes is about three‐fold higher than wild type (Figure 5D). In contrast, the average velocity in class I oocytes may be a little faster but is not significantly different from wild type. MyoV is highly perdurant in the *Drosophila* oocyte, such that a maternal contribution can cause a range of phenotypes (e.g., Krauss et al. 2009). In this case, because the motion fell into two classes, as opposed to a continuum, and because class I is statistically indistinguishable from wild type, we speculate that class I represents cases in which a second crossover event occurred between the *didum* and ovo^D^ loci, creating heterozygotes instead of nulls. Any difference between wild‐type and class I may be due to the presence of MyoV‐motor from the remaining copy of *didum* ^154^. With this in mind, we focused on class II for further analysis. Confirming that the differences were due to loss of MyoV, both streaming and coordination were reduced to wildtype levels upon expression of MyoV‐GFP in *didum* ^154^‐GLCs (Figure 5D,E).

**FIGURE 5:**
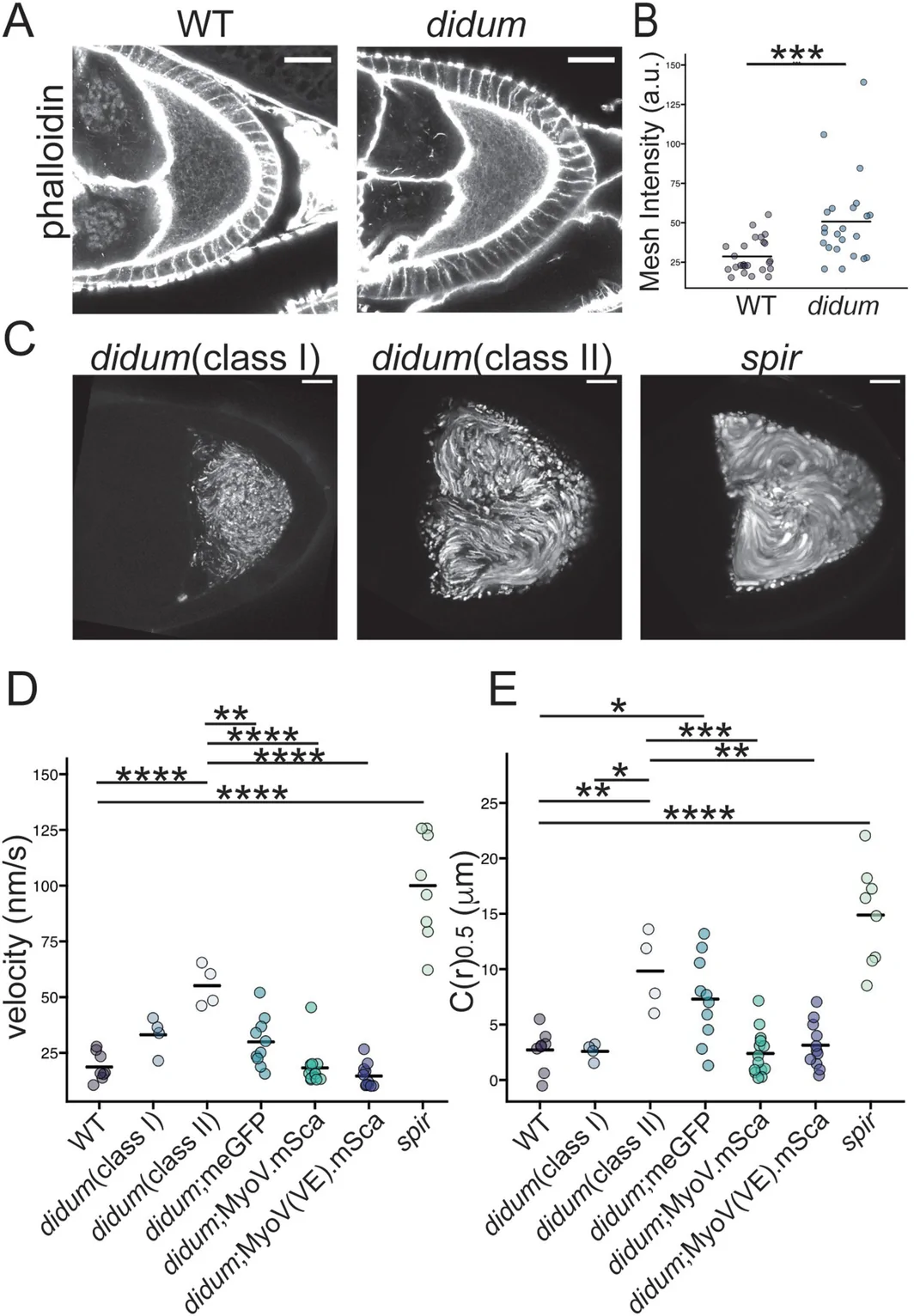
A streaming intermediate. (A) Actin mesh intensity in *didum*‐GLCs is higher than what is observed in wild type. (B) Quantification of average mesh intensities. ***p = 0.00058. (C) Comparison of time lapse projections of yolk granule autofluorescence in stage 9 oocytes (1 image/5 s for 5 min). (D) Streaming velocity is increased in *didum*‐GLC(class II) relative to wildtype and rescued by expression of MyoV‐mScarlet or MyoV(VE)‐mScarlet. (E) Correlation of motion is increased in *didum*‐GLC(class II) relative to wild type and rescued by expression of MyoV‐mScarlet or MyoV(VE)‐mScarlet. Statistical analyses are one‐way ANOVAs followed by Tukey Post Hoc tests. Significance codes: ****p < 0.0001, ***p < 0.001, **p < 0.01, *p < 0.05. Scale bars = 20 μm.

While the velocity of *didum* ^154^‐GLCs (class II) is faster than wild type it is also significantly slower than typical premature fast streaming, such as that observed in a *spir*‐null (Figure 5D). Slower streaming is presumably due to the presence of the actin mesh, which hinders motion, if not as effectively as it does in wild type oocytes. Thus, we have identified a new streaming state and a new phenotype caused by the loss of MyoV.

### 2.5 Loss of Spir‐MyoV Binding Does Not Disrupt Oogenesis

In order to test the significance of the direct interaction between Spir and MyoV to oogenesis, we generated fly lines expressing proteins that do not interact, based on our in vitro data (Figure 3). Specifically, we generated fly lines for expression of the binding mutant UASp‐SpirB(LAIA)‐GFP to compare with our existing UASp‐SpirB‐GFP fly line, and we generated fly lines for expression of UASp‐MyoV‐mScarlet and its binding mutant UASp‐MyoV(VE)‐mScarlet. First, we compared the localizations of SpirB‐GFP and SpirB(LAIA)‐GFP. SpirB(LAIA)‐GFP localization in the *spir*‐null background is comparable to the localization of wild‐type SpirB‐GFP in the *didum* ^154^‐GLC background (Figure 6A vs. 2F). There is significantly less Spir‐LAIA at the cortex relative to the oocyte (1.6 ± 0.3 vs. 1.1 ± 0.3, *p* = 0.006) and only rarely are punctae detected, as was the case for SpirB‐GFP in the *didum* ^154^‐GLC background (Figure S2D,E). The similarity between these two phenotypes suggests that MyoV‐binding influences wild‐type Spir localization. Despite the altered localization of Spir(LAIA)‐GFP, the actin mesh and slow streaming are restored in a *spir*‐null background (Figure 6B,C). Consistent with these observations, expression of SpirB‐GFP or SpirB(LAIA)‐GFP, driven by capu‐GAL4, rescues the fertility (egg hatching) rate of *spir*‐null flies (*spir* ^1^/*spir* ^KG00320^ fertility = 0%) to approximately 55% (similar to our published data for SpirB‐GFP driven by nanos‐GAL4 (Quinlan 2013)).

**FIGURE 6:**
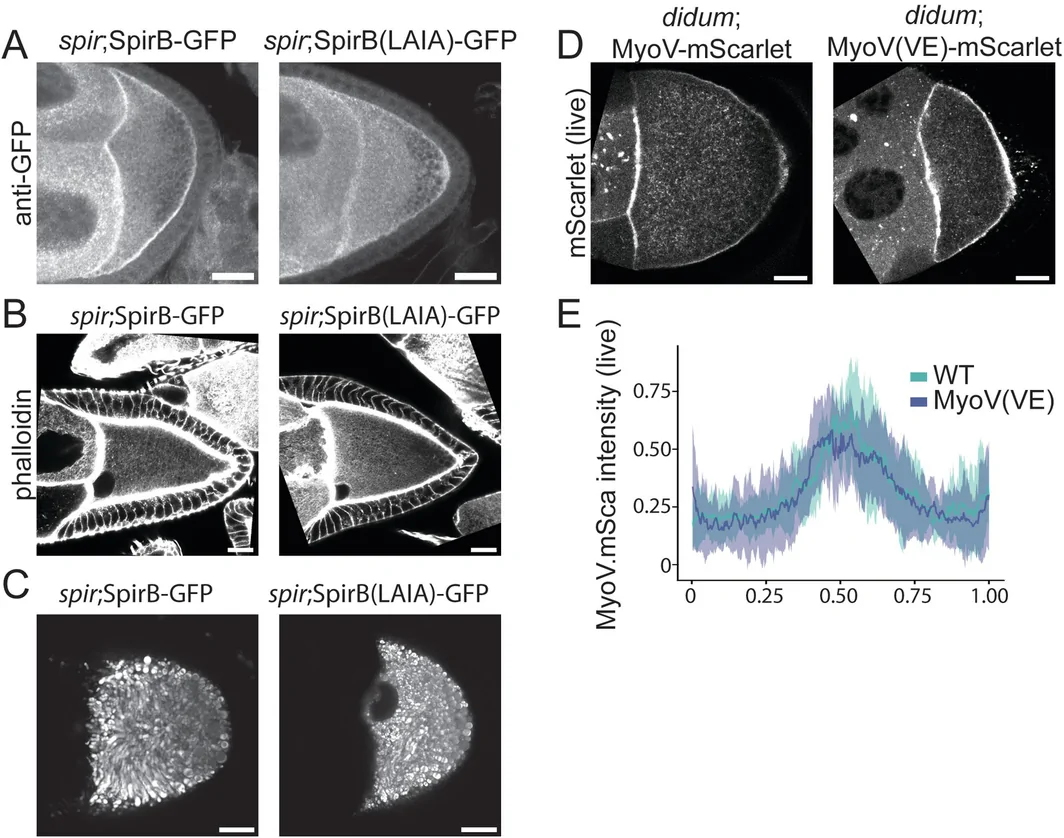
Loss of Spire‐MyoV binding does not disrupt oogenesis. (A) Binding mutant SpirB(LAIA)‐GFP is less enriched at the oocyte cortex and punctae are not detected. (See Figure S2E for comparison of relative intensities). (B) Actin mesh intensity does not differ in a *spir* background, whether rescued with SpirB‐GFP or the binding mutant. (C) Time lapse projections of yolk granule autofluorescence in stage 9 oocytes (1 image/5 s for 5 min) reflect slow streaming. (D) Localization of MyoV(VE)‐mScarlet is indistinguishable from wildtype MyoV in a *didum*‐GLC background. (E) Quantification of cortical mScarlet in live oocytes shows posterior enrichment for both MyoV alleles (MyoV(VE) *n* = 10 oocytes from two flies, WT *n* = 7 oocytes from three flies). Scale bars = 20 μm.

We then expressed either wildtype MyoV‐mScarlet or MyoV(VE)‐mScarlet in *didum* ^*154*^‐GLCs. Both constructs localized to the cortex of the oocyte and were enriched at the posterior, as seen with MyoV‐GFP (Figure 6D,E). In addition, both constructs were sufficient to rescue streaming velocity and coordination (Figure 5D,E). Rescue of fertility was poor but indistinguishable between MyoV‐mScarlet and MyoV(VE)‐mScarlet (both ~30%). This poor fertility result may reflect a difference between the driver and native expression patterns of MyoV. Nonetheless, it reveals no significant biological difference between the two constructs in our assay. Altogether, these results suggest that mutations sufficient to disrupt SpirB‐MyoV binding in vitro have little impact on oogenesis.

## 3 Discussion

### 3.1 Do Spir and MyoV Interact in *Drosophila* Oocytes?

While the data demonstrate that *Drosophila* Spir and MyoV can interact, their direct interaction is not required for oogenesis. The altered localization of Spir in *didum* ^154^‐GLCs at the cortex suggests that MyoV influences Spir localization (Figure 2F). The similarity between the localization of Spir in *didum* ^154^‐GLCs and that of Spir(LAIA) in a wildtype background supports this role for MyoV via direct binding (Figures 2F and 6A). There is a high probability that a third protein, such as a Rab‐GTPase, complexes with Spir and MyoV. However, this putative third protein is not sufficient to overcome the loss of Spir/MyoV binding to drive wild type Spir localization. In fact, while compiling this manuscript, a report that mammalian Rab11a and Spire‐2 together activate MyoVa was published (Yao et al. 2024). Consistent with what we found with *Drosophila* proteins (Figure 3C), Yao et al., observed that mammalian Spir alone only weakly activates MyoV. This is in direct contrast to melanophilin, which is sufficient to activate MyoV ATPase activity (Li et al. 2005). It will be interesting to study how interaction with Rab 11 (or another Rab) influences MyoV and Spir in the oocyte.

So why is the mesh present and fertility unaffected in Spir(LAIA) flies if localization is altered? Previous experiments with a Spir isoform that lacks the membrane binding mFYVE domain (SpirD) demonstrated that cortical enrichment, and presumably localization on punctae, is not required for mesh formation (Dahlgaard et al. 2007; Quinlan 2013). Interestingly, in both SpirD‐expressing oocytes and in *didum* ^154^‐GLCs, the mesh density is higher than wild type (Figure 5A) (Quinlan 2013). Similarly, Spir‐positive vesicles are lost in mouse oocytes expressing Rab11a‐S25N, but actin mesh density is increased (Holubcová et al. 2013). Perhaps MyoV and/or membrane binding play a regulatory role, decreasing Spir activity. Regardless, the simplest explanation of our observations is that while Spir and MyoV are both essential for oogenesis and have overlapping localizations and roles in the oocyte, their direct interaction is not necessary for actin mesh formation or polarity establishment.

### 3.2 What Does Intermediate Streaming Tell Us?

Spir and MyoV influence the actin mesh in distinct ways, as revealed by the changes in mesh dynamics of heterozygotes (Figure 4E). In *spir*/+ oocytes, the dynamics were variable but, on average, they decreased (tau was longer). Decreased dynamics, in this case, may reflect a decrease in actin assembly because Spir levels are lower. While mesh dynamics of *didum*/+ oocytes did not significantly differ from those of wild‐type flies, the dynamics in *spir/didum* transheterozygotes were comparable to those of wild‐type oocytes. Thus, decreasing MyoV levels compensated for decreased Spir levels. We propose that MyoV crosslinks actin filaments throughout the mesh. In the *spir/didum* background, decreased crosslinking could permit increased mesh dynamics compensating for low levels of actin assembly due to decreased Spir.

An apparently contradictory observation comes from the mouse oocyte. Upon expression of a dominant negative MyoVb tail, mesh dynamics are lost (Holubcová et al. 2013). A major difference between fly and mouse oocytes is the localization of microtubules. The large majority of microtubules in the mouse oocyte are in the meiotic spindle. In contrast, the fly oocyte is essentially filled with microtubules that emanate from the cortex. Kinesin‐driven movement of microtubules in the fly oocyte increases flows in mid‐oogenesis and drives fast streaming in late oogenesis. These flows increase actin‐mesh movement in wildtype oocytes (Drechsler et al. 2017). Consistently, in preliminary experiments, mesh dynamics were greatly decreased in *didum* ^154^‐GLCs when the fly egg was treated with colchicine (not shown).

An interesting difference between fly and mouse MyoV is their processive movement. Most MyoV motors studied take many steps before falling off actin filaments in vitro, making them well‐designed to transport cargo in cells (Wong and Weisman 2021). In contrast, *Drosophila* MyoV is not a processive motor, at least in vitro (Bonafe and Sellers 1998). As such, its ability to drive the movement of the actin mesh, as seen in the mouse oocyte, may be limited (Schuh 2011). While collections of MyoV could potentially support processive movement by *Drosophila* MyoV, we speculate that crosslinking/anchoring may be its dominant role here. Consistent with this idea, data indicate that posterior enrichment of osk mRNA and Staufen is not mediated by MyoV transport. Instead, MyoV at the posterior captures and/or anchors these polarity factors (Lu et al. 2020, 2018). Thus, MyoV's role in mouse and *Drosophila* oocytes may be similar, that is, crosslinking actin filaments, but appears distinct due to their different levels of processive movement.

Next we consider why streaming velocity is intermediate, and not faster, in *didum* ^154^‐GLCs. In *didum* ^154^/+ oocytes streaming velocity is not increased. The motion, however, is more ordered. In *didum* ^154^‐GLCs, the correlation is higher than that of the heterozygote. In addition, the streaming velocity increases. These observations are consistent with the idea that MyoV crosslinks/stabilizes the actin mesh in *Drosophila* oocytes, assuming less crosslinking allows self‐organization of flows, as observed in the limit of fast streaming. The model does not explain streaming rates. A straightforward explanation is that a threshold of crosslinking is sufficient to prevent increased streaming velocity. If there are other crosslinkers in the mesh, loss of crosslinking due to a decrease of MyoV levels does not cross that threshold; in the absence of MyoV, the threshold is crossed.

An alternate explanation comes from considering the mechanism underlying the transition to fast streaming. Fast streaming is driven by kinesin‐mediated motion of microtubules. We know that either removing the mesh (e.g., latrunculin treatment) or inactivating dynein results in premature fast streaming (Emmons et al. 1995; Serbus et al. 2005). These data suggest that the force of kinesin‐driven microtubule motion is sufficient (in the absence of active dynein) to disrupt the wildtype actin mesh (Serbus et al. 2005). If mesh crosslinking by MyoV resists mesh disruption, intermediate streaming in the absence of MyoV suggests that more than baseline force levels (e.g., activation of more kinesin or inhibition of dynein) are typically required to displace the mesh prematurely.

The need for increased force to drive premature streaming in the presence of the actin mesh, however, does not necessarily indicate that activation of kinesin or inhibition of dynein is part of the wildtype process of mesh removal. In fact, kinesin‐driven flows are not necessary for mesh disassembly (Dahlgaard et al. 2007). Spir and Capu protein levels decrease dramatically around the stage 10 transition, suggesting that they cease to build the mesh after mid‐oogenesis (Quinlan 2013). Prematurely removing the mesh when it is still being built could be accomplished by reorganization, that is, pushing filaments out of the way, even if they continue to exist. Pushing the filaments necessitates overcoming the effective viscosity created by the mesh. The effective viscosity may be lower when MyoV is absent but the increased mesh density in *didum* ^154^‐GLCs would push the needle in the opposite direction. Thus, the mesh in stage 9 *didum* ^154^‐GLCs could partially resist kinesin‐driven flows, resulting in intermediate streaming velocity.

## 4 Materials and Methods

See Table S2 for commercial reagents and their sources.

### 4.1 *Drosophila* Stocks

Fly lines created for this paper include pUASP::MyoV‐mScarlet, pUASp::MyoV(VE)‐mScarlet, and pUASp::SpirB(LAIA)‐GFP. For MyoV, amino acid numbering is consistent with Uniprot ID A1Z6Z8, and for Spir, amino acid number follows Uniprot ID Q9U1K1 and CG10076 isoform B. In all cases, the gene was inserted using the Drosophila Gateway Vector System (Akbari et al. 2009). The open reading frame, including the fluorescent tag, was amplified via PCR using primers to add attB recombination sites and cloned into pDONR221 using BP clonase. Constructs were subsequently swapped with LR clonase into pPW‐attB1 (we added a phiC31 attB1 site into pPW). BestGene Inc. integrated plasmids into the attP2 position on chromosome 3 (RRID:BDSC_8622). White‐eyed flies (*w* ^1118^, RRID:BDSC_3605) were used as wild‐type controls. Additional fly lines used in this paper are listed in Table 1.

**TABLE 1:** Fly lines.

Name	Source	Identifier (e.g., RRID)	References
Df(2L)Exel6046	BDSC	BDSC_7528	(Parks et al. 2004)
nanos‐GAL4‐vp16	BDSC	BDSC_4937	(Van Doren et al. 1998)
FRT 42, ovo^D^/CyO,T(1;2)OR64	BDSC	BDSC_4434	Donor: Jonathan Wasserman, MRC Laboratory of Molecular Biology
spir^1^	BDSC	BDSC_5113	(Schüpbach and Wieschaus 1991)
spir ^MI05646^	BDSC	BDSC_43877	(Nagarkar‐Jaiswal et al. 2015)
spir ^KG00320^	BDSC	BDSC_13087	Donor: Berkeley Drosophila Genome Project; Donor's *Source:* Gary Karpen, Salk Institute for Biological Studies
didum ^KG04384^	BDSC	BDSC_14094	Donor: Berkeley Drosophila Genome Project; Donor's Source: Hugo J. Bellen, Baylor College of Medicine
didum ^154^ hsFLP; didum^154^, FRT 42B/CyO, and UASp‐MyoV‐GFP	A. Ephrussi		(Krauss et al. 2009)
capu ^HK3^	T. Schüpbach		(Manseau and Schupbach 1989)
w[*]; P{w[+mC] = His2Av‐EGFP.C}2/SM6a	BDSC	BDSC 24163	
pUASg‐SpirC‐3xHA	FlyORF.ch	F001174	(Bischof et al. 2013)
pUASg‐Rab11‐3xHA	FlyORF.ch	F001035	(Bischof et al. 2013)
GFP‐Stau2/Cyo; TM2/TM6B	I. Palacios		
UASp‐SpirB‐GFP			(Quinlan 2013)

### 4.2 GLC Generation

GLCs were generated as previously described (Chou et al. 1993; Krause et al. 2002; Krauss et al. 2009). Briefly, female flies (hsFLP; FRT42B‐*didum* ^154^/CyO; transgene/TM6B) and a corresponding male (hsFLP/Y; FRT42B‐ovo^D^/Cyo;nos‐Gal4‐vp16) were introduced into a fresh food vial for 24 h before being cleared. Approximately 48 h after the introduction of the flies into the vial, the larvae were heat‐shocked in a water bath at 37°C for 1 to 2 h. This heat shock was repeated during subsequent days for two or three heat shocks per vial (see Figure S1 for more information).

### 4.3 Paraformaldehyde Fixation

For Stau staining, a protocol adopted from (Lu et al. 2016) was used. Briefly, ovaries were dissected into cold PBS (140 mM NaCl, 2.7 mM KCl, 1.8 mM KH_2_PO_4_, 10 mM Na_2_HPO_4_, pH 7.4) and transferred to a 1.5 mL microcentrifuge tube. For fixation, the PBS was replaced with 4% paraformaldehyde in PBS (v/v) and rocked on a rotating platform for 15 min. The ovaries were washed four times with PBST (PBS + 0.1% Triton X‐100) and blocked in PBT1 (PBS + 0.1% Triton X‐100 + 5% BSA) for 1–2 h. Ovaries were stained with anti‐Stau antibody (see Table S3 for all antibody details) diluted in PBT1 (1:50) and allowed to incubate overnight at 4°C on a rotating platform. The ovaries were washed four times with PBT1. The appropriate secondary antibody diluted in PBT1 (1:500) was added. If used, Hoechst 33342 was added to the secondary antibody staining solution at a final concentration of 1 μg/mL. After 2–3 h of staining, the ovaries were washed three times with PBT1, twice with PBS, and then mounted in Prolong Gold and sealed with a coverslip.

### 4.4 Hexane‐Based Fixation

For HA and GFP staining, a protocol adapted from (Cooley et al. 1992) was used. Ovaries were dissected into cold PBS and transferred to a 1.5 mL microcentrifuge tube. The PBS was removed and 100 μL of Devit buffer (50 μL of PBS + 250 μL of 7.2% paraformaldehyde in water) and 600 μL of heptane were added simultaneously. The tube was gently inverted to mix and the sample was incubated for 5 min on a rotating platform. The ovaries were washed twice with PBS, twice with PBT3 (PBS + 0.3% Triton X‐100 + 0.5% BSA), then incubated with PBT3 three times for 10 min each. The ovaries were stained with primary antibody (anti‐GFP and/or anti‐HA tag, both at 1:500 dilution in PBT3) overnight and the appropriate secondary antibody (1:500 dilution in PBT3) for 2–3 h. Samples were washed and prepared for imaging as described above.

### 4.5 Phalloidin Staining of Actin Mesh

Phalloidin staining of the actin mesh was performed as described (Dahlgaard et al. 2007; Quinlan 2013). Briefly, ovaries were dissected directly into 10% PFA in PBS (v/v). After washing with PBS, the ovaries were stained with 2 μM phalloidin in PBS + 0.3% Triton X‐100 for 25–30 min before washing and mounting in ProLong gold and sealing with a coverslip. The oocytes were imaged the next day because phalloidin binding to *Drosophila* actin is not as stable as in many other species.

### 4.6 Microscopy

Most confocal microscopy images were acquired on a Leica TCS SPE with an ACS APO 63× 1.30 Oil objective. Images of MyoV‐mScarlet (Figure 6D) were acquired on a LSM780 with a EC Plan‐Neofluar 40×/1.3 Oil DIC M27 objective. AiryScan images (Figure 2A,B,D,E,E′,E″ and Figure S2A–C) were acquired on a LSM880 platform with a Plan‐Apochromat 63×/1.4 Oil DIC M27 objective, with no frame averaging.

#### 4.6.1 Mesh Quantification

Ovaries from *didum* ^154^‐GLCs (heat‐shocked hsFLP; FRT42B‐didum^154^/ovo^D^), *didum* ^154^/+ (hsFLP; FRT42B‐didum^154^/CyO), and wildtype flies (expressing histone‐GFP) were dissected into 10% PFA. To help control for the time each sample spent in PFA, the flies were dissected in three batches: first *didum* ^154^‐GLCs (Set A), then *didum*/+, and finally *didum* ^154^‐GLCs again (Set B). This approach allowed for assessing any effects of overfixation of the mesh, as differences between *didum* ^154^‐GLCs Set A and Set B could be attributed to fixation time. To control for differences in staining intensity, within each batch, dissections alternated between a *didum* ^154^‐GLCs or *didum*/+ fly and a wildtype Histone2B‐EGFP fly. As the wildtype ovaries received the same treatment as the *didum* ^154^‐GLCs ovaries, differences in the staining intensity of the wildtype ovaries across the three sets could be attributed to biological variability and variation in staining procedure between the three separate tubes of fly ovaries.

The mesh was stained with 2 μM Alexa Fluor 488 phalloidin. Within each set, wildtype oocytes could be distinguished from *didum* ^154^‐GLCs or *didum* ^154^/+ oocytes by the nuclear Histone2B‐EGFP fluorescence (most prominent in nurse cells and follicle cells), which did not interfere with mesh quantitation. Normalized mesh intensity was obtained by drawing the largest rectangle that could fit within the oocyte (taking care to only capture the actin mesh and to avoid cortical actin) and dividing the mean fluorescence intensity by the area of the rectangle.

#### 4.6.2 MyoV Quantification

Images of live oocytes expressing fluorescently labeled MyoV were acquired. The cortex was traced from anterior to posterior back to anterior of the oocyte using the freehand line tool (line width = 10) in FIJI (Schindelin et al. 2012). The resulting plot profile was processed with a series of Python scripts. The first Python script normalized the highest *y*‐values (MyoV intensity) to 1. The second Python script then averaged the intensity values within 400 bins of *x*‐values (distance). This approach was taken to account for different oocyte sizes. The third Python script was used to calculate the average and standard deviation across multiple datasets from the same genotype and graph them. Scripts are available at the following URL: https://github.com/jong2ucla/MyoVSpir.

#### 4.6.3 SpirB‐GFP Quantification

Images of fixed oocytes expressing fluorescently labeled SpirB were acquired. Line scans (20 pixels wide) were drawn across the cortex to include the oocyte, follicle cells, and background. For visualization, intensities were normalized to the signal in the follicle cells, which should reflect background tissue staining given that SpirB expression was driven in the germline only (Figure S2D,E). Cortical intensity was defined as the average of the top four intensities. The oocyte intensity was defined as the average of 20 data points (~5 μm) taken ~5 μm away from the cortex. Cortical intensity relative to oocyte intensity was calculated to account for normal variation in expression level.

#### 4.6.4 Ooplasm Analysis

Cytoplasmic streaming velocities were determined from confocal images using the particle image velocimetry lab (PIV lab) MATLAB package generated by William Thielicke (Thielicke and Sonntag 2021; Thielicke and Stamhui 2014). Regions of interest (ROIs) were drawn around the oocyte and the background was masked so only the area containing yolk (the oocyte cytoplasm) was interrogated. The FFT window deformation algorithm, a direct Fourier transformation correlation with multiple passes and deforming windows, was used. Interrogation areas of 30 px with a 15 px step (50%) were used, decreasing to 20 and 10 px. Limits were selected to refine data, and PIV lab was permitted to interpolate missing data based on surrounding values. The vector information was extracted from the flow pattern and the mean streaming velocity was determined for each oocyte analyzed. Mid and late‐stage oocytes were analyzed separately, using this method.

Output files of vector information were processed in custom Correlation analysis software, 2DVelocityCorrelation (https://github.com/LiamABailey/2DVelocityCorrelation), based on (Dombrowski et al. 2004; Ganguly et al. 2012). For each frame of PIV data, a correlation function was determined and then averaged to generate the correlation for the oocyte. For each oocyte we determined the maximal radius at which the vectors were half correlated and plotted the data in R using ggplot2. Statistics such as one‐way ANOVA with Tukey–Kramer post hoc test were used to determine significance between genotypes were performed in R. See graphs for information about *p*‐values generated.

#### 4.6.5 Mesh Dynamics Analysis

Images of the actin mesh labeled with UtrnCH‐GFP were acquired from stage 9 oocytes, collecting 1 frame every 10 s. Motion was analyzed with FIJI using the Image CorrelationJ plugin.

### 4.7 Recombinant Protein Cloning and Purification

Cloning of C‐terminally GST‐tagged Spire 1–490 and His‐Capu CT was described previously (Vizcarra et al. 2011). N‐terminally His‐tagged MyoV tail constructs (1411–1800, WT and V1477E) were cloned into pET‐15b backbone vectors using standard cloning procedures. Other Spir‐GST constructs (1–520, LAIA point mutants) were cloned similarly into a pET‐20b backbone vector. Point mutations were introduced using site‐directed mutagenesis.

Expression: Rosetta II bacteria were grown in Terrific Broth media with 100 mg/L ampicillin at 37°C until an optical density at 600 nm of 0.6–0.9, cooled to 18°C for 1 h, and induced with 250 μM IPTG for 18 h at 18°C. The bacteria were pelleted by centrifugation, washed with ice‐cold PBS, flash‐frozen in liquid nitrogen, and either immediately used for purification or stored at −80°C.

All purification steps were carried out at 4°C or on ice. Thawed cells were diluted with lysis buffer (50 mM sodium phosphate, 1 mM beta‐mercaptoethanol, 300 mM NaCl, pH 8.0) with 1 mM PMSF and 2 μg/mL DNase I. Lysis was performed by passing the cells through a microfluidizer two or three times. Lysates were cleared by centrifugation at 20,000 rcf for 20 min, and the clarified lysates were incubated with metal‐affinity TALON resin for 1 h (using 2 mL of resin slurry for every 1 L of cell culture). Spir‐GST constructs were also purified using TALON resin; at amino acid 205, Spir contains a series of histidine residues that bind to the resin. The TALON resin was washed with 20 column volumes of lysis buffer, 20 column volumes of wash buffer (lysis buffer at pH 7.0), and the proteins were eluted with elution buffer (wash buffer + 200 mM imidazole).

The TALON eluates were pooled and dialyzed into 10 mM Tris (pH 8.0) and 1 mM DTT, then loaded onto a MonoQ anion exchange column. Spir‐GST constructs were eluted using 0–500 mM NaCl, while other constructs were eluted over 50–250 mM KCl. Pooled fractions from MonoQ columns were dialyzed into 10 mM Tris (pH 8.0) and 1 mM DTT, and their protein concentrations were assessed from their measured absorbance at 280 nm and theoretical ε_280_ coefficients. The proteins were dialyzed into a buffer of 50% glycerol and 50% dialysis buffer, aliquoted, flash‐frozen in liquid nitrogen, and stored at −80°C until use.

The His‐MyoV‐tail TALON eluates were dialyzed into 50 mM MES (pH 6.0), 150 mM NaCl, and 1 mM DTT and loaded onto a MonoS cation exchange column. MyoV constructs were eluted off with a gradient of 150–500 mM NaCl. Pooled fractions were dialyzed into 50 mM MES (pH 6.0), 150 mM NaCl, and 1 mM DTT, and then treated like other proteins.

Full‐length MyoV was purified via the baculovirus‐*Sf9* protein purification system as previously described (Lu et al. 2020). Briefly, cDNA encoding MyoV was inserted into a modified pFastBac1 vector containing a C‐terminal FLAG tag. Recombinant baculoviruses were generated according to the manufacturer's protocol (Thermo Fisher Scientific). For protein production, *Sf9* insect cells were co‐infected with recombinant baculovirus encoding the MyoV heavy chain, *Drosophila* calmodulin, and *Drosophila* cytoplasmic myosin light chain (*Mlc‐c*). Cells were pelleted after 53 h of expression.

Cell pellets were resuspended in extraction buffer containing 500 mM NaCl, 10 mM MgCl_2_, 1 mM EGTA, and 10 mM MOPS, pH 7.2, supplemented with freshly added 2 mM ATP, 0.1 mM DTT, and tablets of protease inhibitor cocktail (Roche). Cells were lysed by sonication, and the lysates were clarified by centrifugation at 48,000*g* for 30 min. The supernatant was incubated with anti‐FLAG resin (Sigma) for 1 h at 4°C. The resin was subsequently washed twice with master buffer (500 mM NaCl, 10 mM MgCl_2_, 1 mM EGTA, 100 μM Leupeptin, 500 μM PMSF, and 10 mM MOPS, pH 7.2) and bound proteins were eluted by the addition of 300 μg/mL FLAG peptide. Eluted proteins were dialyzed overnight against buffer containing 10 mM MOPS, 500 mM NaCl, 0.1 mM EGTA, 2 mM MgCl_2_, and 1 mM DTT, pH 7.2. Myosins were further concentrated by low‐speed centrifugation (4000*g*, 15 min) using Amicon filter units (Millipore Sigma) and flash frozen in liquid nitrogen for future use.

### 4.8 Co‐Immunoprecipitation

For binding experiments using proteins from fly lysates, the ovaries of approximately 20 flies of each genotype (expressing meGFP, MyoV‐GFP, SpirC‐3xHA, or Rab11‐3xHA) were dissected into cold PBS. The PBS was removed with a pipette and replaced with 130 μL of cold lysis buffer (50 mM HEPES pH 7.4, 150 mM KCl, 1 mM EDTA, 1 mM MgCl_2_, 10% glycerol, 0.5 mM DTT, protease inhibitor cocktail, and 0.2% NP‐40 detergent). The ovaries were lysed with a plastic pestle, incubated on ice for about 10 min, then cleared by centrifugation at 13,000*g* for 5 min. Ten microliter of the lysates (representing about 20% of the amount used for each binding reaction) was reserved as a loading control. The lysates were added in equal volumes (about 50 μL of lysates for both HA‐ and GFP‐tagged protein) to 20 μL of magnetic HA‐beads and allowed to bind at 4°C for 2.5 h with gentle rocking. The beads were washed four times (5 min per wash) with lysis buffer and the proteins were eluted from the magnetic beads by boiling in SDS‐PAGE sample loading buffer. The Western blot was incubated with GFP and HA‐tag antibodies (both at 1:1000 dilution) in PBST + 0.5% BSA overnight and with the secondary antibodies (both at 1:10,000 dilution) for 1–2 h before imaging on a LICOR Odyssey imager.

For binding experiments using purified recombinant proteins, equimolar concentrations of proteins diluted in cold PBS and 1/20th of the input were reserved as a loading control. Samples were incubated with glutathione resin and allowed to bind at 4°C for 2.5 h with gentle rocking. The resin was washed with PBS three times and the proteins were eluted by boiling in SDS‐PAGE sample loading buffer.

### 4.9 Mass Photometry

Single‐molecule mass photometry was performed as previously described (Liu et al. 2021) using a Refeyn One^MP^ mass photometer. Briefly, microscope coverslips (#1.5 24 × 50 mm and 24 × 14 mm, Fisher Scientific) were cleaned sequentially with isopropanol and water, then dried using a stream of clean nitrogen. Flow chambers were assembled by stacking coverslip pairs and sealing them with double‐sided tape. MyoV, Spire, or a mixture of both was introduced into the chamber in buffer containing 150 mM NaCl, 2 mM MgCl_2_, 0.1 mM EGTA, and 10 mM MOPS, pH 7.2. Data were collected over a ~3 μm × 10 μm field of view at an acquisition rate of 1 kHz for 100 s. All measurements were carried out at room temperature (~24°C). Images were processed using manufacturer‐supplied software (Refeyn, UK). The conversion between molecular mass and interferometric contrast was calibrated using protein standards of known molecular weight. For each sample, a mass distribution histogram was generated and fitted with a Gaussian curve.

### 4.10 MyoV ATPase Assays

Steady‐state actin‐activated MyoV ATPase activities were measured using a SpectraMax ID3 microplate reader (Molecular Devices Inc) at 37°C in buffers containing 10 mM MOPS, pH 7.2, 1 mM ATP, 50 mM NaCl, 2 mM MgCl_2_, and either 0.1 mM EGTA or 0.1 mM free CaCl_2_. MyoV (20 nM), actin and Spir were added at concentrations indicated. The buffer also contains an NADH‐coupled ATP‐regenerating system including 40 units/mL lactate dehydrogenase, 200 units/mL pyruvate kinase, 200 μM NADH and 1 mM phosphoenolpyruvate. ATP hydrolysis rates were determined by measuring the decrease in absorbance at 340 nm due to NADH oxidation. Data were collected using Softmax Pro 7.1 software (Molecular Devices) and plotted in GraphPad Prism 7.0.

### 4.11 Yeast Two‐Hybrid

As described for fly line generation, the *Drosophila* Gateway Vector System was used to clone DNA constructs for the yeast two‐hybrid assay. MyoV fragments of interest were cloned into the pDEST‐pGBKT7 (bait) plasmid and the Spir constructs were cloned into the pDEST‐pGADT7 (prey) plasmid. The MyoV plasmids were transformed into the Y2HGold yeast strain, and the Spir plasmids were transformed into the Y187 strain.

To perform the yeast two‐hybrid assay, 20 μL of each prey Y2HGold or bait Y187 strain of log‐phase yeast culture was added to 100 μL YPD in a 96‐well plate and incubated for 24 h at 30°C with shaking. The mating culture (3–4 μL) was plated onto double‐drop out (DDO; −Leu, −Trp) plates and grown for 5 days at 30°C. Colonies were replica plated onto the following plates: DDO, quadruple drop out (QDO; −Leu, −Trp, −Ura, −Ade), DDO + 20 mg/mL X‐α‐Gal + 200 ng/mL aureobasidin A, and QDO + 20 mg/mL X‐α‐Gal + 200 ng/mL aureobasidin A. The plates were incubated for 5 days at 30°C and photographed using an iPhone. A positive bait‐prey interaction was identified when the colony was blue and could grow on QDO + 20 mg/mL X‐α‐Gal + 200 ng/mL aureobasidin A plates.

## Author Contributions

**Joseph Y. Ong:** conceptualization, investigation, formal analysis, methodology, writing – original draft. **Hannah M. Bailey:** investigation, formal analysis, methodology, writing – review and editing. **Emma Carley:** investigation. **Michelle Panzica:** investigation. **Merin Rixen:** investigation. **Neil Billington:** investigation, writing – review and editing. **Rong Liu:** investigation, writing – review and editing. **Margot E. Quinlan:** conceptualization, investigation, formal analysis, funding acquisition, writing – review and editing.

## Funding

This work was supported by the National Institute of General Medical Sciences (R01GM096133 and T32GM007185), National Science Foundation (DGE‐1650604) the National Heart Lung and Blood Institute (L'Enfant Biomedical Fellowship).

## Conflicts of Interest

The authors declare no conflicts of interest.

## Supporting information


**Data S1:** Supporting information.

## Data Availability

The data that support the findings of this study are available from the corresponding author upon reasonable request.
