# Supplementary material for: The Influence of Drosophila Spire and Myosin V During Mid‐Oogenesis Is Independent of Their Direct Interaction
Source: Cytoskeleton (Hoboken). 2025 Dec 8;83(10):779–94. doi: 10.1002/cm.70080 (PMC13648917; doi:10.1002/cm.70080)
Supplement: Supplementary file 1 [file CM-83-779-s001.docx]

**Supplementary Information for:**

The influence of Drosophila Spire and MyosinV during mid-oogenesis is independent of their direct interaction

Joseph Y. Ong^1^, Hannah M. Bailey^1^, Emma Carley^1^, Michelle Panzica^1^, Merin Rixen^1^, Neil Billington^2^, Rong Liu^2^, Margot E. Quinlan^1,3^

^1^Department of Chemistry and Biochemistry, University of California, Los Angeles, CA 90095

^2^Department of Biochemistry and Molecular Medicine, West Virginia University, Morgantown, WV 26506

^3^Molecular Biology Institute, University of California, Los Angeles, CA 90095

*Corresponding Author: quinlanme@g.ucla.edu

Key Words: Spire, Myosin V, Drosophila, Oogenesis, Actin

The authors declare no conflict of interest.

# Supplemental Tables

Table S1. Hatching rate of heterozygotes

| genotype | eggs laid | %hatched |
| --- | --- | --- |
| spir^1^/+ | 182 | 96.7 |
| didum^KG04384^/+ | 200 | 85.5 |
| didum^154^/+ | 216 | 95.8 |
| spir^1^/didum^KG04384^ | 356 | 91.0 |
| spir^1^/didum^154^ | 436 | 93.3 |

Table S2: Critical reagents

| Commercial reagent | Manufacturer | Catalog/ID Number |
| --- | --- | --- |
| Anti-HA-tag mAb-Magnetic Beads (Monoclonal Antibody) | MBL International | M132-11 |
| Precision Plus Protein Dual Color Standards, 500 µL | BIORAD Life Science Group | #1610374 |
| SDS-PAGE Molecular Weight Standards, Broad Range | BIORAD Life Science Group | #1610317 |
| 4–20% Mini-PROTEAN® TGX™ Precast Protein Gels, 15-well, 15 µL | BIORAD Life Science Group | #4561096 |
| Pierce™ Protease Inhibitor  Tablets, EDTA-free | Thermo Fisher Scientific | A32965 |
| Alexa Fluor™ 488 Phalloidin | Thermo Fisher Scientific | A12379 |
| Alexa Fluor™ 647 Phalloidin | Thermo Fisher Scientific | A22287 |
| Modified Matchmaker Gold Yeast Two-Hybrid system | Laboratory of N. Rusan, NIH |  |
| X-α-Gal | Takara Bio | 630463 |
| Aureobasidin A | Takara Bio | 630466 |
| Deoxyribonucleic acid sodium salt from salmon testes | Sigma Aldrich | D1626-250MG |
| Gateway™ pDONR221 Vector | Invitrogen | 12536017 |
| Gateway™ BP Clonase™ II Enzyme mix | Invitrogen | 11789020 |
| Gateway™ LR Clonase™ II Enzyme mix | Invitrogen | 11791020 |

Table S3: Antibodies

| Antibody | Manufacturer | Catalog Number/Identifier |
| --- | --- | --- |
| HA-Tag Antibody (C29F4) | Cell Signaling Technologies | #3724 |
| GFP Antibody | Aves Labs | GFP-1010  Antibody registry ID: AB_2307313 |
| IRDye® 800CW Donkey anti-Chicken IgG (H + L), 0.5 mg | LI-COR Biotechnology | 926-32218 |
| IRDye® 680RD Donkey anti-Rabbit IgG (H + L), 0.5 mg | LI-COR Biotechnology | 926-68073 |
| Fluorescein (FITC) AffiniPure Donkey Anti-Chicken IgY (IgG) (H+L) | Jackson ImmunoResearch Laboratories | 703-095-155 |
| Cy™5 AffiniPure Donkey Anti-Rabbit IgG (H+L) | Jackson ImmunoResearch Laboratories | 711-175-152 |
| Staufen antibody | Laboratory of Chris Q. Doe, University of Oregon | N/A |

# Supplemental Figures

**Supplemental Figure 1. Germline clones (GLCs).** A) So-called Dominant Female Sterile stocks that include ovo^D^ and the recombinase pair FLP/FRT were used with an FRT42B-*didum^154^* line, generously provided by the Ephrussi lab, to build *didum*-GLCs (Chou et al., 1993; Krauss et al., 2009). Egg chamber development arrests early in ovo^D^ heterozygotes and homozygotes. Flipase expression (FLP) and subsequent recombination at FRT sites results in four possible genotypes. Egg chambers homozygous for *didum^154^* (m in the figure) will be the only surviving samples at stage 9 and beyond. The egg chamber is not completely devoid of MyoV because a small amount is made by the heterozygous mother and contributed to the egg chamber precursor cell before recombination is complete. No full-length MyoV is detected by Western blotting in embryos, confirming absence of the wildtype gene in GLCs. (See Krauss et al., 2009 Figure S1E.) Figure from Bloomington Drosophila Stock Center website. B) Phenotypic differences in the original report (Krauss et al., 2009) and here are consistent with expression of a stable product from *didum^154^* in GLCs, which includes most of the motor domain, a putative dominant negative.

**Supplemental Figure 2. Supplement to figure 2.** A) Image from figure 2D separated by channel. B) A control egg chamber that does not express 3xHA-SpirC or MyoV-GFP, prepared on the same day and imaged with the same microscope settings. C) The same image in B (left) with the brightness greatly increased (~20x) to show that a sample is present. (Channels are the same in each column.) D) Yellow lines shown were scanned (20 pixels wide) for quantification of cortical enrichment in egg chambers expressing SpirB-GFP in WT or *didum*-GLC backgrounds. Signals were normalized to follicle cell intensity for comparison. E) Line scans of egg chambers expressing SpirB-GFP or SpirB(LAIA)-GFP. Ratios of cortex intensity/oocyte intensity are given. Upper plot are traces from images in D. F) Live imaging reveals that posterior cortical enrichment of MyoV-GFP is lost in most *capu* flies (*capu*_1, n = 11 oocytes from 6 flies) but oocytes from one fly had wild type MyoV-GFP distribution (*capu*_2, n = 7 oocytes from 1 fly). Timelapse overlays of yolk granule fluorescence demonstrates that the *capu*-null results in premature streaming in both cases. Traces denoted *capu_*# were capu[HK3];nos::GAL4>UASp::MyoV-GFP flies. G, top) Quantification of GFP intensity along the cortex reveals enrichment of MyoV-GFP at the posterior of the oocyte in WT and *capu*/+ backgrounds. *capu*^HK3^/+ flies were *capu*^HK3^/CyO;nos::GAL4>UASp::MyoV-GFP (n = 8 oocyte from 3 flies) G, bottom) MyoV-GFP enrichment at the posterior is lost in *capu*_1 oocytes but only slightly lowered in *capu*_2 oocytes. Scale bars = 20 μm

**Supplemental Figure 3. Direct interaction between Spir and MyoV.** A) Diagram of constructs used in yeast two-hybrid assay. The Spir plasmids were transformed into the Y187 strain and the MyoV plasmids were transformed into the Y2HGold yeast strain. See Methods for more details. B) Results demonstrate that SpirB residues 490-520 are necessary and sufficient to bind the MyoV-GTD. n/d indicates the interaction test was not performed. C) Anti-HA immunoprecipitation from ovary lysates. Lysate of control ovaries that do not express SpirC-3xHA or Rab11-3xHA was mixed with MyoV-GFP containing lysate. MyoV-GFP does not co-IP with anti-HA beads alone.

**Supplemental Figure 4.** A) Gel and mass photometry of purified full length MyoV with light chains. The mass histogram has a major peak at 544 kDa, corresponding to a MyoV dimer. (HC, Myosin V heavy chain; calmod, calmodulin; ELC, essential light chain.) B) Gel and mass photometry of purified Spir520.Two major mass species were detected: 58 kDa and 102 kDa, accounting for approximately 50% and 40% of total measurements, respectively. The 58 kDa is close to the predicted mass of a Spir520 (60 kDa), thus it should represent monomeric molecules. The 102 kDa species may represent a dimer. C) Mass photometry of a MyoV-Spir520 mixture (30 nM MyoV incubated with 100 nM Spir520 for 1hr at room temperature). We detected the mass corresponding to Spir520 (overlapping with the lower-mass impurities from MyoV sample) and dimeric MyoV, but did not see Gaussian distributions that match the mass of a MyoV-Spir520 complex at any stoichiometric ratio. D) Pyrene-actin assembly assay shows that polymerization kinetics are the same for 25 nM Spir520 when MyoV-GTD is added.

**Supplemental Figure 5. Intermediate streaming does not change Stau localization.** A) Stau appears enriched at the posterior cortex of fixed wild type, heterozygous (*spir*/+ or *didum*/+), and *spir/didum* transheterozygous stage 9 oocytes. B) Time lapse projection of yolk granule autofluorescence shows intermediate streaming in a stage 9 *didum*/+ oocyte expressing GFP Stau (1 image/5 sec for 5 minutes). Stau appears enriched at the posterior cortex of the live oocyte. Scale bars = 20 μm
